# The impact of vitreous floaters on quality of life: a qualitative study

**DOI:** 10.1186/s41687-025-00934-w

**Published:** 2025-08-18

**Authors:** Jarinne E. Woudstra-de Jong, Sonia S. Manning-Charalampidou, Johannes Hans R. Vingerling, S. J. Feike Gerbrandy, Konrad Pesudovs, Jan J. Busschbach

**Affiliations:** 1 Rotterdam Ophthalmic Institute, Eye Hospital Rotterdam, Schiedamse Vest 160, Rotterdam, 3011 BH The Netherlands; 2 Department of Psychiatry, Section Medical Psychology and Psychotherapy, Erasmus Medical Centre, Rotterdam, The Netherlands; 3 Department of Vitreoretinal Surgery, Eye Hospital Rotterdam, Rotterdam, The Netherlands; 4 Department of Ophthalmology, Erasmus Medical Centre, Rotterdam, The Netherlands; 5 Oogheelkundig Medisch Centrum Amstelland / Amsterdam Eye Hospital, Diemen, The Netherlands; 6 School of Optometry and Vision Science, Medicine and Health, University of New South Wales, Sydney, Australia

**Keywords:** Vitreous floaters, Patient perspective, Quality of life, Patient-reported outcome measurement, Ophthalmology

## Abstract

**Background:**

In this study we explore the quality-of-life impact of vitreous floaters, and identify all possible issues that may be relevant to a person experiencing floaters, with the aim to define the content of a floaters-specific patient-reported outcome measure, which can help determine the disease burden and benefit of treatment for a patient with floaters.

**Methodology:**

Semi-structured telephone interviews were conducted with patients with floaters. The topic guide was based on an extensive literature review and expert opinion. Interview transcripts were coded into all possible quality-of-life issues that may be relevant to a person experiencing floaters. We grouped these into quality-of-life domains and subdomains, using an earlier framework of quality of life in other ophthalmic diseases.

**Results:**

We categorized the patients’ reports (*N* = 44) into 11 quality-of-life domains: activity limitations, visual symptoms, ocular symptoms, general symptoms, health concerns, emotional well-being, social well-being, mobility issues, economic impact, inconveniences, and the need to use coping strategies.

**Conclusions:**

Vitreous floaters result in specific quality-of-life issues that have not been adequately measured with previous patient-reported outcome measures. When compared to other eye conditions, three previously unidentified issues stand out among patients with floaters: the effects of different lighting conditions, intermittent vision impairment caused by eye movements, and the importance of seeking understanding and support from healthcare providers. Eye care clinicians should recognize these issues when evaluating patients with floaters. The present patient-driven determination of relevant quality-of-life issues can guide initiatives in floaters-specific patient-reported outcome measures.

**Supplementary Information:**

The online version contains supplementary material available at 10.1186/s41687-025-00934-w.

## Abstract (plain language summary)

Many people perceive moving spots in their vision (vitreous floaters), but not everyone is bothered by them. The impact of floaters is difficult to assess with clinical tests. Previous studies have used questionnaires or a small number of interviews to investigate quality of life, but these studies may not cover all relevant quality-of-life issues. With 44 interviews, we found that vitreous floaters can have a unique and previously unidentified impact on the quality of life of individuals with this eye condition, such as difficulties in daily activities, concerns about work, being bothered by bright light, and wanting their concerns to be taken seriously. Eye care clinicians should recognize these issues when evaluating a person who experiences floaters. To help weigh the quality-of-life impact of floaters and their treatments for each individual, a floater-specific questionnaire is helpful, but only if it is relevant, valid, and developed with patient input. The findings from this study can inform the development of a new floaters-specific questionnaire that covers a more complete range of relevant quality-of-life issues.

**Supplementary Information:**

The online version contains supplementary material available at 10.1186/s41687-025-00934-w.

## Background

When the clear filling of the eye contains any opacities, these can be visible to a person as vitreous floaters: moving spots in their vision. Many people perceive vitreous floaters, with self-reported prevalence levels reaching 87% of the general population, but not everyone is bothered by them [1–5]. After ocular pathology has been ruled out by an ophthalmologist, patients are reassured that the floaters cannot cause harm to their vision, and that most people get used to them. For some, the floaters persist, and can cause considerable psychological burden and impact on quality of life [1, 5]. It is unclear why some people tolerate floaters while others do not.

It can be difficult for ophthalmologists to decide whether to intervene, based solely on visual acuity and symptoms. Ophthalmological treatments exist to attempt to remove the opacities, but there is debate whether the symptoms of patients justify the potential risks of treatment [6]. Quantitative evaluations of floaters exist, such as quantitative ultrasonography and contrast sensitivity [7, 8], but these do not inform on the experienced personal impact of floaters. Information about quality of life can be helpful in assessments of disease burden, treatment decisions, and evaluations of treatment effects.

To help ophthalmologists weigh the quality-of-life impact for each individual patient, a floaters-specific patient-reported outcome measure (PROM) would be helpful, but only if it is relevant, valid, and developed with comprehensive patient consultation. Unfortunately, current PROMs used for vitreous floaters were developed without comprehensive consultation of patients and reports on validity and reliability are minimal, resulting in suboptimal measures of quality of life [9]. Qualitative research is a necessary starting point for PROM development to ensure content validity, i.e. that the measurement adequately reflects the patients’ perspective [10–12]. 

Only one explorative qualitative study exists to our knowledge that describes the impact of floaters, such as difficulty in activities, work, and social life, and disease and treatment-related concerns, resulting in a variety of emotional reactions and different coping strategies [13]. Existing floaters-specific PROMs mostly cover difficulty in activities and symptoms, but neglect to measure other important quality-of-life issues [9]. The qualitative study provides valuable information on quality of life in patients with floaters; however, as it was not aimed specifically to investigate quality-of-life issues for PROM development and only covered 11 interviews, it potentially missed relevant issues.

The current study is the first comprehensive qualitative study to our knowledge on the quality-of-life impact of floaters and potential treatment. People suffering from floaters may find the overview of potential quality-of-life impact caused by floaters insightful and recognizable, validating their own experience. The purpose of this study is to gather information and report on the patient experience to inform future PROM development. We aim to uncover all potential quality-of-life issues that could be relevant to a person experiencing floaters and/or receiving treatment for floaters, and that should be included in a floaters-specific PROM. This information can help clinicians to identify patients who are heavily impacted by floaters when considering potential treatment. The results can also be used to explore the content validity of existing floaters-specific PROMs and/or help define the content of future PROMs.

## Methods

We explored quality-of-life issues through semi-structured telephone interviews with people who have been diagnosed with vitreous floaters. We followed international guidelines for PROM development and qualitative research [10–12]. 

To assure sufficient variance in the sample, we selected participants with different ages, levels of education, visual acuities, and stages of treatment through purposive sampling. We included people with floaters in one or both eyes, as diagnosed and confirmed by an ophthalmologist, that were willing and able to participate in a telephone interview. We included participants that did not want or need treatment, and people who were on the waiting list for treatment or had undergone Nd:YAG laser vitreolysis and/or pars plana vitrectomy surgery. People were excluded for inability to understand and speak Dutch, cognitive impairment, and any visually significant comorbid ophthalmic disorder. We also excluded people whose floaters were caused by vitreous hemorrhage or uveitis. We did not exclude people with comorbid or previous cataracts.

Participants were initially recruited between January and April 2022 from vitreoretinal clinics in the Rotterdam Eye Hospital, an eye center in The Netherlands. Other potential participants were identified from a list of new floaters diagnoses at this hospital in 2021. After recommendations of journal reviewers, additional participants were recruited in January 2024 from the OMC Amstelland / Amsterdam Eye Hospital, one of the few centers in The Netherlands that offers Nd:YAG laser vitreolysis to people with floaters. Eligible patients were given a participant information flyer and consent form. Participants received a gift card of €25 after the interview.

Upon receiving the signed consent form, participants were called for a telephone interview by JEWdJ [researcher and a trained interviewer]. The interviews were semi-structured and followed a topic guide (supplementary materials). The topic guide was based on existing literature on quality-of-life issues in vitreoretinal conditions; [14, 15] our review on floater-specific PROMs [9], and expert opinion [SSMC and JRV are vitreoretinal specialists, JJB and KP are experts on quality-of-life measurement; all reviewed and provided input to the topic list]. The topic guide included 11 quality-of-life domains that are established in other eye disorders: symptoms (visual, ocular, and general), activity limitation, mobility, health concerns, emotional and social wellbeing, convenience, economic impact, and coping [14–25]. 

The central question during the interviews was: “*What is it like to live with floaters?*”, in line with phenomenological theory for the development of PROMs, as operationalized by Brédart et al. [10, 26] The interviews followed the participant’s story over time: from the first onset of floaters, the current experience, to the expected future experience. The interviewer used general questions to find quality-of-life issues that were not previously conceptualized, e.g., “*Can you tell me about the floaters? How did/do they impact you?*”, and only moved to specific inquiries if a pre-conceptualized domain had not been mentioned by the participant, e.g., for social well-being: “*How did/do the floaters impact your social life?*”. Interviews were recorded and transcribed verbatim and coded by JEWdJ using the software NVivo [27]. 

JEWdJ and SSMC reviewed all mentions of quality-of-life issues to verify if any issue did not fit under one of the previously conceptualized domains, but found that all mentioned issues could fit into the 11 domains. Information from the transcripts was coded into segments, grouped into sub-themes, and clustered into the 11 pre-conceptualized quality-of-life domains; for example, the segment ‘*I cannot read small print’* was coded as “Difficulty reading small print” and grouped into the sub-theme “Difficulty reading” with other codes relating to reading, which was then clustered into the quality-of-life domain “Activity limitation”. We coded text segments related to outcomes information, i.e. quality-of-life issues, related to the impact of floaters and/or effectiveness of treatment. We did not code text segments that were solely related to quality of care, e.g. satisfaction with hospital facilities, as quality-of-care issues are measured with a patient-reported experience measure, not with an outcome measure.

Data analysis and sampling occurred concurrently to determine when saturation was reached, defined as no new quality-of-life issues emerging from 5 consecutive interviews [28–30]. No new quality-of-life issues emerged after 39 interviews, so we continued for 5 more interviews to verify data saturation. In total, we interviewed 44 participants (interview length: 7 to 44 min). The interviews were conducted between March and October 2022, and in January and February 2024.

## Results

Among 120 potential participants approached, 57 signed informed consent (response rate: 47.5%) and 44 participated. (Table 1; *N* = 44). Most participants were diagnosed with floaters in both eyes (79.5%) and had received treatment for their floaters (70.5%). The median visual acuity of the worst eye was 90% or 20/160 Snellen. We found 646 unique quality-of-life issues related to vitreous floaters and/or treatment, and coded these into sub-themes (Tables 2 and 3). We were able to categorize all issues into the 11 established domains: visual symptoms, ocular symptoms, general symptoms, activity limitations, mobility problems, inconveniences, economic impact, social wellbeing, emotional wellbeing, health concerns, and coping.

**Table 1:** Socio-demographic and clinical characteristics of the vitreous floaters study population (*N* = 44)

Age at time of interview	
Mean ± Standard deviation	65.1 ± 9.4 years
Range	28.6 to 78.6 years
*Gender*	
Female	18 patients (40.9%)
*Residence*	
Same city as hospital	7 patients (15.9%)
Same province as hospital	19 patients (45.5%)
Different province as hospital	17 patients (38.6%)
*Marital status*	
Married/Partnership	30 patients (68.2%)
*Education level*	
Primary or high school education	6 patients (13.6%)
Secondary vocational education	15 patients (34.1%)
Higher professional education or university	23 patients (52.3%)
*Employment status*	
Working	22 patients (50.0%)
*Visual acuity (better eye)*,* decimal (Snellen)*	
Median	1.0 (20/200)
Range	0.7 to 1.5 (20/100 to 20/16)
*Visual acuity (worse eye)*,* decimal (Snellen)*	
Median	0.9 (20/160)
Range	0.3 to 1.5 (20/63 to 20/16)
*Time interval between floaters diagnosis and interview*	
< 3 months	3 patients (6.8%)
3 months − 2 years	26 patients (59.1%)
> 2 years	15 patients (34.9%)
*Laterality*	
Bilateral	35 patients (79.5%)
*Lens status of the affected eye*	
Pseudophakic at time of diagnosis	28 eyes (31.8%)
*Treatment*	
Phacovitrectomy	18 surgeries (14 patients, 31.8%)
Vitrectomy	10 surgeries (8 patients, 18.2%)
YAG laser vitreolysis	48 sessions (15 patients, 34.9%)
On waiting list	7 patients (15.9%)
Not on waiting list	6 patients (13.6%)
Post-treatment complications*	
Yes	9 patients (20.5%)
Ocular comorbidity (visually inconsequential)**	
Yes	15 patients (34.9%)
Medical history***	
Yes	22 patients (50.0%)

Footnotes:

* Post-treatment complications included cystoid macular edema (3), cataract (1), high intraocular pressure (3), retinal detachment (1), and epiretinal membrane (1)

** Ocular comorbidities included high myopia (6), retinal defect (4), amblyopia (2), epiretinal membrane (2), blepharitis (1), macular edema (1) and strabismus (1)

*** Medical comorbidities included hypertension (8), obesity (3), asthma (2), cancer (2), chronic obstructive pulmonary disease (2), hypercholesterolemia (2), mental health disorder (4), transient ischemic attack (2), cardiac arrhythmia (1), diaphragmatic hernia (1), epilepsy (1), tinnitus (1), and vasectomy (1)

**Table 2:** Examples of quality-of-life issues mentioned in interviews with patients with vitreous floaters (*N* = 44)

Quality/of/life domain	Examplary quality-of-life issues	Exemplary quote
Visual symptoms	Seeing black spots	We were on a vacation and I suddenly got these black, well, it almost looked like tiny tadpoles in my eye. And some days they were gone, and then they would appear again. And I found it a bit scary, actually. (Female, 58 years old, 6 months after floaters diagnosis in one eye)
	Vision changing between clear and blurry	It was like seeing a water drop. And for the entire day, you have some moments when you see clearly, and then this kind of veil appears […]. It is extremely bothersome. (Female, 77 years old, waiting for surgery of her second eye)
Ocular symptoms	Dry eyes, Eye pain	I never fully understood the consequences of the surgery. The consequence I now have experienced is that my eye hurts enormously, because it is dry. (Male, 64 years old, 6 months after surgery)
	Eye strain	I have to look in the distance to, well, let my eyes recover from straining, so that they can relax for a while. I do think I have to strain my eyes a lot. (Female, 48 years old, 2 months after surgery)
General symptoms	Headaches	I get these weird headaches. [..] If I watch television, after half an hour my vision starts getting weird and a bit blurry. And I could continue to watch, but then I really have to take a painkiller. (Female, 70 years old, 2 months after surgery)
	Fatigue	Especially in the evenings, I am tired, and I am just tired in my eyes, in my head. And I think: pff, I have had enough for today. (Female, 70 years old, 5 months after floaters diagnosis in both eyes)
Activity limitations	Difficulty reading a book	When reading a book, some parts are just missing. And that is so annoying. Because you constantly have to search and move the book. And at times when I am really into the story, I have to quit reading. […] I have to rest my eye and cannot read. So devouring a book like I could, I cannot do that anymore. (Female, 58 years old, 8 months after floater diagnosis in both eyes)
	Difficulty looking side to side	And I also have difficulty when I, I work with computer screens, so I have two screens in front of me. And when I look from the one screen to the other screen, then yes, yes I am bothered by the floaters. (Male, 59 years old, 2 months after floaters diagnosis in one eye)
Mobility issues	Difficulty stepping on and off curbs	Of course, you are limited. You walk carefully, trying to balance. Because you, well, your vision moves. And you are inclined to miss a step, for instance, when going down the stairs, with simple things. A small curb, where you think: did I step over it? Or did I step on it? And that is a strange experience. (Female, 62 years old, 6 weeks after surgery)
	Difficulty walking down the stairs	I also always have difficulty walking stairs, walking down the stairs. […] Because I cannot see things well, you know. I cannot see depth very well. (Female, 61 years old, 2.5 years after floaters diagnosis in both eyes)
Inconveniences	Wating to get used to the floaters, Weighing the risks and benefits of surgery	[The ophthalmologist said:] ‘You have two healthy eyes, and if you have surgery, there is a risk.’ I get that. I told him that I had really thought it through well. Every surgery has a risk. But this [the floaters] is really getting annoying though. And the longer I wait, the older I get. And then surgery is even more difficult. But fine, I told him I would wait for a little while longer to see if it gets better or if I will get used to it. Because that’s the point: you might get used to it. (Male, 69 years old, has recently been approved for surgery)
	Having to pause during activities	But with reading a book […], part of the page is missing. And that is so annoying, because you sit down. And you have to search [for the words] and move the book, when I am in the middle of the story. Yes, I have to stop reading then. (Female, 58, 2 months after diagnosis with floaters in one eye)
Economic impact	Not being able to work, Having to switch type of job	Because of the work that I do, I really need my eyes, have needed my eyes, I should say, because I just cannot do some types of jobs anymore. And so the consequences, in that sense, are quite big for me. It is all about being able to see really well, and seeing small details really well. I really have to do precision work. (Female, 62 years old, 2.5 years after floaters diagnosis in both eyes)
	Working more slowly	I repair microelectronics, which is quite detailed work, even when using magnification tools […]. It was hard, I was struggling, so I worked a lot more slowly, because I constantly had to make sure that the floaters were out of the way. Which is also why I went to the doctor with the request to fix [my vision]. (Male, 69 years old, 2 months after surgery of his second eye)
Emotional wellbeing	Feeling unlike self	My life has changed so much that I almost do not feel like myself anymore. (Female, 61 years old, after 3 sessions of laser vitreolysis)
	Feeling frightened by sudden appearance of floaters	We have a display with notifications in the bus, and I could not read it anymore […] When a floater blocks your vision, you see nothing for a short moment, and that scares you every time. (Female, 62 years old, 1 month after surgery of her second eye)
Social wellbeing	Feeling misunderstood	It is just unpleasant. In daily life it [my vision] really impacts a lot, and there are not many people who get that. Because they say: ‘Oh, I also have this or that’. Yes, but you have no idea what my vision is like, you know. (Female, 64 years old, 1.5 years after surgery)
	Difficulty describing floaters to other people	No, it is, I also noticed, when I explained it to friends, family, when the first floater appeared. Well, you cannot describe it accurately. No, that is very difficult. (Male, 78 years old, 9 months after surgery)
Health concerns	Getting the ophthalmologist to understand	I wish the doctor could see through my eyes, see what I see, and experience what I experience. (Male, 70 years old, 5 months after floaters diagnosis in both eyes)
	Concerns about surgery, Moving by mistake during surgery	I chose for a general anesthetic. Yes, because then I cannot sneeze during the surgery, or at least, the chance of me moving is a lot smaller. […] You don’t want the doctor to slip up because I moved. (Male, 57 years old, 1.5 months after floaters diagnosis in both eyes)
Coping	Persisting to find treatment	Other seventy-year olds might say: ‘If that happens to my eye, well, then I’ll accept it. I will just sit on the couch more and be less active. And I won’t do something about it’. Well, I am simply not wired that way. (Male, 70 years old, 2 years after surgery)
	Blinking, Moving eyes	You keep your eye very still and try to blink less. And well, then they [the floaters] float to the front of your vision again. So I have to blink for some time, and try to get them out of the way. So that is, well, quite annoying actually. (Male, 56 years old, 5 months after floater diagnosis in both eyes)

**Table 3:** Overview of quality-of-life domains and related sub-themes from interviews with patients with floaters (*N* = 44)

Quality-of-life domain	Sub-theme
Activity limitations	Distance vision Driving Exercise / Sports Finding things Free time activities Household tasks Near vision Reading and writing Using screens Visual activities, e.g. seeing color / contrast / depth
Convenience	Related to activity limitations Related to eye drops Related to floaters symptoms, e.g. being distracted Related to glasses / contact lenses Related to hospital visit, e.g. travel distance Related to posturing after surgery Related to waiting
Coping	Emotional regulation, e.g. acceptance Problem avoidance, e.g. ignoring the problem Problem solving, e.g. using sunglasses Seeking help, e.g. visiting healthcare provider
Economic impact	Other costs, e.g. new glasses Treatment-related costs Work
Emotional wellbeing	Anger Anxiety Changed self-image Depression
General symptoms	Fatigue Headaches Related to treatment, e.g. stiff muscles from posturing Sleep problems
Health concerns	Related to future vision impairment Related to getting correct and understandable information Related to risks and complications of treatment Related to safety, e.g. falling Related to treatment, e.g. getting surgery and anesthesia
Ocular symptoms	Discomfort Pain Red eyes Tired eyes / eye strain
Social wellbeing	Getting support Getting understanding Going out Socializing
Visual symptoms	Blurry vision Difference between image from both eyes Distorted vision Flashes Floaters Problems with lighting conditions, e.g. photosensitivity Related to tamponade, e.g. seeing gas bubble

### **Visual symptoms** (134 quality-of-life issues, 20.6% of 646 quality-of-life issues identified in total)

Floaters were described as spots, dots, flies, worms, flakes, threads, specks, strings, dark clouds, spider webs, greasy smudges, or hazes. For example, participants described their vision ‘*as if there are tadpoles swimming in my eye*’ or ‘*as if my eye is water where plastic bags are floating through*’. The floaters could be transparent or opaque, with different sizes and locations in their visual field, appearing suddenly or growing gradually worse (‘*clumping together’)*. When moving their eyes, participants saw the floaters move. Because of the floaters, their vision changed constantly, alternating between clear and blurry vision, resembling ‘*what you see when you resurface after being under water*,* you cannot see well for a short time*’ or *‘the phase of turning a page*,* where you don’t see the book for one short moment*,* and then you see everything crisp and clear again.*’ Many participants also mentioned seeing flashes at the onset of the floaters.

Some participants reported seeing floaters more in the dark, others experienced them more clearly against a light background. Many participants experienced photosensitivity, where seeing directly into the light was difficult, as it caused ‘*shadows*’ cast by the floaters. Participants had to wear sunglasses or a hat and kept their back towards the sun and other bright lights. Adapting to light after being in the dark was difficult, for instance when coming out of a tunnel when driving.

Participants also reported visual symptoms related to treatment. Air tamponade after vitrectomy caused distorted or obstructed vision, ‘*as if I’m looking through oil*’, which would ‘*slosh’* whenever they moved, until the ‘*water level dropped*’ with clear vision above the air bubble. One person distinctly remembered seeing the floater disappear (‘*being sucked out of my eye’*) during surgery under local anesthesia.

### **Activity limitations*** (123 quality-of-life issues*, *19.5%)*

Not all participants reported difficulties caused by floaters, but many did. Driving was difficult, mostly under circumstances with poor visibility (i.e. in the dark, mist or rain) or in bright light. Participants were startled by sudden floaters, mistaking them for another vehicle or person, which led some to stop driving or drive only short, familiar routes. Concentrating and focusing their eyes was also difficult, especially when reading or using the computer. Reading was particularly difficult because of saccadic eye movements, which made the floaters appear in the center of their vision (‘*they float to the reading part of my vision’)* and obstruct parts of the page or words. Participants consciously had to keep their eyes as still as possible. Similarly, they had difficulty in ‘*looking from one person to another person in a group conversation*’, ‘*switching from looking in the distance to checking the car dashboard when driving*’, and ‘*following a ball during a television sports match*’. Other activities that were affected by floaters were depth perception, watching television or movies in theatre, finding items in the grocery store, and doing detailed tasks, such as typing, crocheting, or writing.

### ***Health concerns****(90 quality-of-life issues*,* 13.9%)*

Initially, participants worried about the cause of the floaters and possible treatment options. Some worried about going blind, thinking the floaters might be a warning sign for a retinal detachment. They wanted to be taken seriously and feel understood by the ophthalmologist; some participants felt they had to convince the ophthalmologist that they needed treatment: ‘*If they [the ophthalmologists] don’t know what’s bothering you*,* how will they be able to help you?’*. They also worried about the effectiveness and safety of treatment: that the floaters might return, possible damage to the retina, and getting cataract. Participants also had safety concerns, such as causing an accident in traffic while driving or falling when walking or cycling.

### ***Inconveniences****(62 quality-of-life issues*,* 9.6%)*

Floaters themselves were experienced as very inconvenient, causing distractions. Participants had to stop what they were doing to wait for the floaters to disappear. Many participants did not like waiting for several months until they might get used to the floaters but wanted a solution directly. If surgery was an option, being on an elective surgery waiting list felt inconvenient, requiring them to postpone plans until the surgery date was known. The weeks after surgery were inconvenient: sleeping with an eye cap, positioning, travel restrictions, instilling eye drops, and obstructed vision because of gas or air tamponade. Other inconveniences were: the travel distance to the hospital, waiting in the hospital for their appointment, undergoing eye examinations, and seeing a different ophthalmologist at each appointment.

### **Emotional wellbeing** (62 quality-of-life issues, 9.6%)

Participants felt more irritable, moody, and impatient because of the constant floaters. Prevalent emotions were frustration, anger, anxiety and depression. Activity limitations led to feeling insecure or handicapped. As floaters from vitreous liquefaction are caused by aging, participants felt ‘*confronted with getting older*’. When treatment was not an option, participants reported feelings of hopelessness and discouragement. Many participants were satisfied and relieved after treatment; some were disappointed.

### **Ocular symptoms** (50 quality-of-life issues, 7.7%)

Floaters caused eye strain for several participants. After treatment, some experienced dry, red, and painful eyes, ocular movement problems, a swollen or hanging eye lid, discomfort from stitches, and a feeling of pressure on the eyes.

### ***Coping strategies****(46 quality-of-life issues*,* 7.2%)*

Participants blinked or moved their eyes to try to get the floaters out of the way. Others tried to keep their eyes as still as possible, using peripheral vision ‘*to see around the floater*’ or trying to ‘*look through the floaters instead of at the floaters’*. Some had to close or cover their eye, because floaters in one eye interfered with binocular vision. Common coping strategies were looking for information about floaters on the internet and contacting a healthcare provider to try and get rid of the floaters. Many used practical strategies, such as changing screen settings to help them read or holding their book closer or further away. Emotional strategies were also frequent: accepting the floaters, trying to ignore them, thinking about nice things, expecting the worst, and taking one day at a time. Many participants tried to enjoy life despite their floaters. Some participants could not accept their floaters, and continued pleading for treatment with their ophthalmologist.

### **Social wellbeing** (24 quality-of-life issues, 3.8%)

Almost all participants had difficulty describing their floaters and how the floaters affected them to other people. They felt that people often forget that floaters are chronic, because ‘*people cannot see from the outside that something is wrong with my eyes*’. Participants often felt misunderstood and lonely, especially when they felt their partner or spouse did not understand them. Many were more dependent on other people, for instance, when having to ask someone to drive them or go with them to the hospital.

### ***Economic impact****(23 quality-of-life issues*,* 3.5%)*

Several participants had to make adjustments to their work, such as taking frequent breaks and working less hours in a day. Many expressed concerns about their ability to work, especially with office jobs where computer use and reading are essential. Two participants had to quit their job, as one could not read anymore and the other was not allowed to drive her work vehicle because of the floaters. Some participants had decided to retire earlier than planned, because of the difficulties at work. For one person, the floaters and treatment caused delay in obtaining her study degree. Participants also had to incur costs, such as paying for treatment, eye checkups, or a new pair of glasses after surgery. It should be noted here that a Nd:YAG laser vitreolysis procedure is not reimbursed by Dutch healthcare insurance, meaning patients have to pay out-of-pocket. Vitrectomy surgery is covered by insurance, and patients are only charged a deductible for surgery.

### ***General symptoms****(20 quality-of-life issues*,* 3.2%)*

The floaters caused fatigue and headaches, which grew worse during the course of the day; many participants had to go to bed earlier than they were used to. Participants also mentioned bodily pain from having to sit still during Nd:YAG laser vitreolysis or lie still during surgery under local anesthetics, or nausea and drowsiness from general anesthesia.

### ***Mobility****(12 quality-of-life issues*,* 1.8%)*

Some participants reported issues with going up or down the stairs, or stepping in and out a bus or train, as the floaters would cause problems with seeing depth. After surgery with tamponade which temporarily obstructs vision in one eye, participants mentioned walking ‘*wobbly’* and tripping easily over bumps and cracks. Several participants had bumped into things because of dilated pupils after eye examinations or treatment.

## Discussion

In this study we interviewed 44 participants with vitreous floaters about their experienced quality of life. We found that floaters, and possible treatment, impacted them in a variety of ways that have not been fully identified before: through experiencing unwanted symptoms, activity limitations, mobility problems, inconveniences, the financial impact, health concerns, impact on emotional and social wellbeing, and the need to use different coping strategies. These qualitative findings increase the understanding of the experienced quality-of-life impact of vitreous floaters.

Compared to quality-of-life issues in other ophthalmic conditions [14–25], three specific issues stand out for people with floaters: the impact of different lighting conditions; intermittent poor vision from eye movements; and the importance of being understood by healthcare providers. A subset of patients with floaters visits multiple ophthalmologists in an attempt to get treatment [31], and often feel like they have to convince the ophthalmologist that the floaters are a serious problem. The decision for treatment depends on different factors, including risks associated with surgery, the surgeon’s experience, opinions within the retina community, and fear of unreasonable patient expectations [32]. It is important that ophthalmologists are aware of patients’ need for recognition, and acknowledge the impact that floaters can have on daily life.

The participants in our study mentioned all issues that are described in a previous exploratory qualitative study with 11 patients with floaters [13]. We again found that floaters can cause many emotional reactions and usage of a variety of different coping strategies. Our study found many additional quality-of-life issues, such as difficulty with reading and driving; concerns about going blind and about getting a complication from treatment; and being startled each time the floaters appear.

The variety of affected quality-of-life domains, and the difficulty to objectively estimate the burden of floaters, argues for the use of a properly developed floater-specific PROM. PROMs can be helpful in diagnostic and treatment evaluation, but only when the content of the PROM is relevant for people experiencing floaters. As we found in a previous literature review [9], all existing PROMs developed specifically for floaters have been developed without comprehensive consultation with patients that are actually affected by floaters. The pooled 85 questions in these current PROMs cover only 13.2% of the 646 quality-of-life issues mentioned by the participants in this study, mostly only related to symptoms and activity limitations. Our study followed international PROM development guidelines and recommendations for qualitative research that require including patients [10–12]. Our findings are well-rooted in the patient voice, thus making them more relevant and useful in measuring the patient experience of floaters. The vast majority (87%) of the issues identified by participants in our study, are new, making this the most comprehensive study of the impact of floaters on quality of life yet. Comprehensive qualitative studies, such as our own, highlight the limitations of PROMs that are developed without considerable patient involvement, and can guide new initiatives for PROM development.

The reported quality-of-life issues can be included in new floaters-specific PROMs and/or be used to verify content validity of existing PROMs. To create a usable PROM, two important conditions need to be met: relevant content and good psychometric quality. The results of our qualitative study can be used for content development and inform on which items to include in a floaters-specific PROM [33]. Next, studies would need to provide evidence for psychometric quality of the PROM in the target population, such as its validity, reliability, and responsiveness [34]. Both of these conditions are important to come to meaningful PROM scores.

One limitation concerns generalizability of our findings, as we only included participants that visited an ophthalmologist for their vitreous floaters, and that reside in the Netherlands. Our research group is currently validating the results with people with self-diagnosed floaters and patients from other countries. Also, we did not compare quality-of-life impact before and after treatment, or between vitrectomy surgery and NdYAG laser vitreolysis. Comparisons are best made with quantitative measures, such as a valid PROM. Such an instrument can be used to answer highly relevant clinical questions, such as differences in the quality-of-life impact between different subgroups, correlations with clinical assessments, and specific treatment effects after different types of treatment.

## Conclusion

Floaters and potential treatment impact quality of life through unwanted symptoms, activity limitations (especially reading and driving), impact on socio-emotional wellbeing, health concerns, economic impact, inconveniences, and the need for coping strategies. This is the first comprehensive qualitative study to our knowledge that identifies and details the many quality-of-life issues that may be relevant to a person experiencing floaters, and that should be included in a floaters-specific PROM. These issues have not been adequately measured with previous PROMs. Ophthalmologists should recognize these specific issues when evaluating patients with floaters, for instance by using a valid floater-specific PROM that is developed with comprehensive patient consultation.

## Supplementary Information

Below is the link to the electronic supplementary material.

Supplementary Material 1

## Data Availability

Data sharing is not applicable to this article as no datasets were generated or analysed during the current study.

## Abbreviations

PROM: Patient-reported outcome measure

## Declarations

### Previous submissions

This submission has not been published anywhere previously and it is not simultaneously being considered for any other publication. It has previously been reviewed and rejected by 5 ophthalmological journals: Ophthalmology Retina (submitted: 28 Aug 2023, rejected: 25 Sep 2023), RETINA (submitted: 5 Mar 2024, rejected: 14 Jul 2024), Eye (submitted: 26 Jun 2024, rejected: 5 Nov 2024), Graefe’s Archive for Clinical and Experimental Ophthalmology (submitted: 14 Nov 2024, rejected: 1 Jan 2025), and Ophthalmic Epidemiology (submitted: 5 Jan 2025, rejected: 24 Feb 2025).
