# Supplementary material for: The impact of vitreous floaters on quality of life: a qualitative study
Source: J Patient Rep Outcomes. 2025 Aug 18;9:102. doi: 10.1186/s41687-025-00934-w (PMC12360991; doi:10.1186/s41687-025-00934-w)
Supplement: Supplementary file 1 — Supplementary Material 1 [file 41687_2025_934_MOESM1_ESM.pdf]

Supplementary material

Topic guide

Semi-structured telephone interview guide to inquire about quality-of-life issues in patients with vitreous floaters.

***Introduction to telephone conversation*:** Good morning/Good afternoon, this is [name interviewer], researcher at the Rotterdam Eye Hospital. You had consented to an interview about your experience with your eye condition. Is that convenient now? (If not, when can I call you back?)

Introduction to the interview: I will first give some information about this conversation:
You have read in the information leaflet that this study concerns your experience with vitreous floaters. We are very curious about the impact the condition has on you. This will help our understanding of what patients with floaters need. In this conversation, I will ask some questions and listen to your answers. There are no right or wrong answers. We especially want to know about your personal experience. That is why we value everything you say as important. Do you have any questions? (If not, we will move on to the questions.)

***Diagnosis*:** You were diagnosed with vitreous floaters in [month] of [year]. Can you take me back in time?

- What did you notice about your eyes and vision at the time?
- How was that for you? What did you feel? What did you think?
- How did you cope with your floaters?
- Did the floaters limit you?
- What was it like for you to be diagnosed?
- What made you decide to (not) have surgery?

***Treatment*:** *Only if the patient had surgery or other treatment* - How was that for you?

- What did you feel? What did you think?
- How was the recovery phase for you?
- What was your vision like, compared with before the treatment?
- What symptoms did you have after the treatment?
- Did you have any expectations of the surgery/treatment? Did the treatment meet your expectations?
- Did you have information about the treatment in advance? What advice did you get? Was the information/advice sufficient for you?
- What did you think of the quality of the treatment?
- Are you satisfied with the treatment?

***Current issues*:** How are you doing now?

- Have the symptoms changed over time?
- Do you have trouble doing certain things?
- Do you ever struggle with your responsibilities/tasks?
- Are there things you can no longer do?
- Did you need to adjust your daily life?

- Do you need help from other people? Do you use tools or make adjustments?
- Do you have trouble with traveling/walking/driving/public transport?
- What was/is the most bothersome thing for you?
- What inconveniences have you experienced? / What inconveniences do you experience as a result of the condition or the treatment? (for example: posturing, recovery, hospital appointments, eye exam, travel, parking)
- What is it like for you to visit the hospital now? How do you feel after you visit?

- How do the floaters/treatment affect...:
    - …your daily life?
    - …your work or other obligations?
    - …your household?
    - …interacting with your partner/spouse/family?
        - Do you experience support from your partner/spouse/family?
        - Do you experience understanding from your partner/spouse/family?
    - …interacting with other people?
        - Do you experience support from the people around you?
        - Do you experience understanding from the people around you?
    - …your hobbies?
    - …how you feel? (e.g. emotions, self-image)
- How do you cope? Do you feel you have control?
- Did the condition or treatment have a financial impact? Did you have to incur costs due to the condition or the treatment? (example: travel, insurance, loss of income)

Future: How do you see the future?

- Do you ever worry? (for example: impact of the condition, loss of vision, impact on other people, your safety, etc.)
- What do you expect from the condition in the future?
- What do you expect from the hospital in the future?

Ending the call**:** That was my last question. Do you have anything to add that is important for me to know?

- If I sum everything up like this: [summary], do you want to add something to that?
- Did I miss something important?
- What would your advice be to another patient with floaters?

Do you have any questions after this conversation?

I would like to thank you very much for your honest answers and for your time.

Useful phrases for during the interview**:**

- *And what else?*
- *That’s interesting. Can you tell more about that?*
- *What do you mean exactly?*
- *Can you give an example?*

How did that happen? / What's the cause of that?
